# Myoepithelioma of the Lung: A Rare Case With Bilateral Pleural Spread and Diagnostic Challenges in a Middle-Aged Female

**DOI:** 10.7759/cureus.100935

**Published:** 2026-01-06

**Authors:** Manoj Mahajan, Mayanka Seth, Manish Seth, Pawan Nikhra, Kavita Gupta, Sunita Nain

**Affiliations:** 1 Department of Medicine and Oncology/Hematology, Pacific Medical College and Hospital, Pacific Medical University, Udaipur, IND; 2 Department of Pathology, Medicentre - Unit of Redcliffe Labs, Udaipur, IND; 3 Department of Radiology, Medicentre - Unit of Redcliffe Labs, Udaipur, IND; 4 Department of Pathology, Pacific Institute of Medical Sciences, Udaipur, IND; 5 Department of Pathology, Pacific Medical College and Hospital, Pacific Medical University, Udaipur, IND; 6 Department of Clinical Pathology, Life Medical Centre, Dubai, ARE

**Keywords:** adenoid cystic carcinoma, bilateral pleural metastasis, epithelioid cells, lung tumor, myoepithelial cells, plasmacytoid cells, pleuro-parenchymal nodules, pulmonary myoepithelioma

## Abstract

Myoepitheliomas are uncommon neoplasms, predominantly arising in the salivary glands, with pulmonary involvement being extremely rare. Primary lung myoepitheliomas account for only a small number of reported cases, while pleural or bilateral presentations are exceptionally uncommon. Their nonspecific radiological appearance often raises suspicion for metastatic disease, necessitating histopathological and immunohistochemical evaluation for accurate diagnosis.

We report a diagnostically challenging case of pulmonary myoepithelioma in a 42-year-old female with a history of treated adenoid cystic carcinoma (ACC) of the buccal mucosa, in which bilateral pleural nodules initially raised strong suspicion for metastatic disease. She remained asymptomatic during follow-up; however, a surveillance computed tomography (CT) scan of the chest revealed multiple bilateral pleuro-parenchymal nodules, the largest measuring 1.1 cm. A CT-guided biopsy demonstrated sheets of tumor cells with round vesicular nuclei, moderate cytoplasm, occasional clear-cell change, and acinar structures containing eosinophilic secretions. No mitoses, necrosis, or hemorrhage were identified. Immunohistochemistry showed positivity for pancytokeratin, smooth muscle actin (SMA), SRY-box 10 (SOX10), p63, and H-Caldesmon, with negative staining for other lineage markers, and a Ki-67 index <2%. These findings confirmed a diagnosis of benign pulmonary myoepithelioma. The patient required no additional therapy and remains under close follow-up.

This case highlights an exceptionally rare presentation of pulmonary myoepithelioma with bilateral pleural involvement - a pattern that can closely mimic metastatic disease - emphasizing the importance of histopathological and immunohistochemical assessment in differentiating benign myoepithelial tumors from metastatic or aggressive pleural lesions.

## Introduction

Myoepithelioma of the lung is a rare neoplasm arising from myoepithelial cells, which are commonly found in salivary glands but can also occur in the respiratory tract. The most common histological types among salivary gland-type tumors are adenoid cystic carcinoma (ACC) and mucoepidermoid carcinoma (MEC), while mixed tumors (ACC and MEC) and myoepithelioma are less common. Myoepithelioma is classified as either benign or malignant, based on cellular atypia, mitotic activity, and invasive potential. Pulmonary myoepitheliomas are infrequent, with only a limited number of cases reported in the literature [1]. They belong to the broader category of salivary gland-type tumors of the lung and share histopathological features with their salivary gland counterparts. Histologically, these tumors consist of spindle, plasmacytoid, clear, or epithelioid cells arranged in a myxoid, hyalinized, or fibrous stroma. Immunohistochemical staining helps to differentiate them from other lung neoplasms [2]. Due to their rarity, the clinical behavior, optimal management, and prognosis of pulmonary myoepitheliomas remain uncertain, necessitating further studies and case reports to better characterize this entity [1-3].

This case gains importance due to its rarity and its distinction from metastatic lesions in a patient with a known salivary gland malignancy. 

## Case presentation

A 42-year-old female with a documented history of ACC of the buccal mucosa, diagnosed in 2021, had been treated with a combination of external beam radiation therapy and systemic chemotherapy. She demonstrated a satisfactory therapeutic response, with resolution of symptoms and no evidence of local or systemic recurrence during subsequent follow-up visits. Since completion of treatment, she remained clinically asymptomatic and continued under routine oncological surveillance, without requiring any additional interventions.

**Figure 1:**
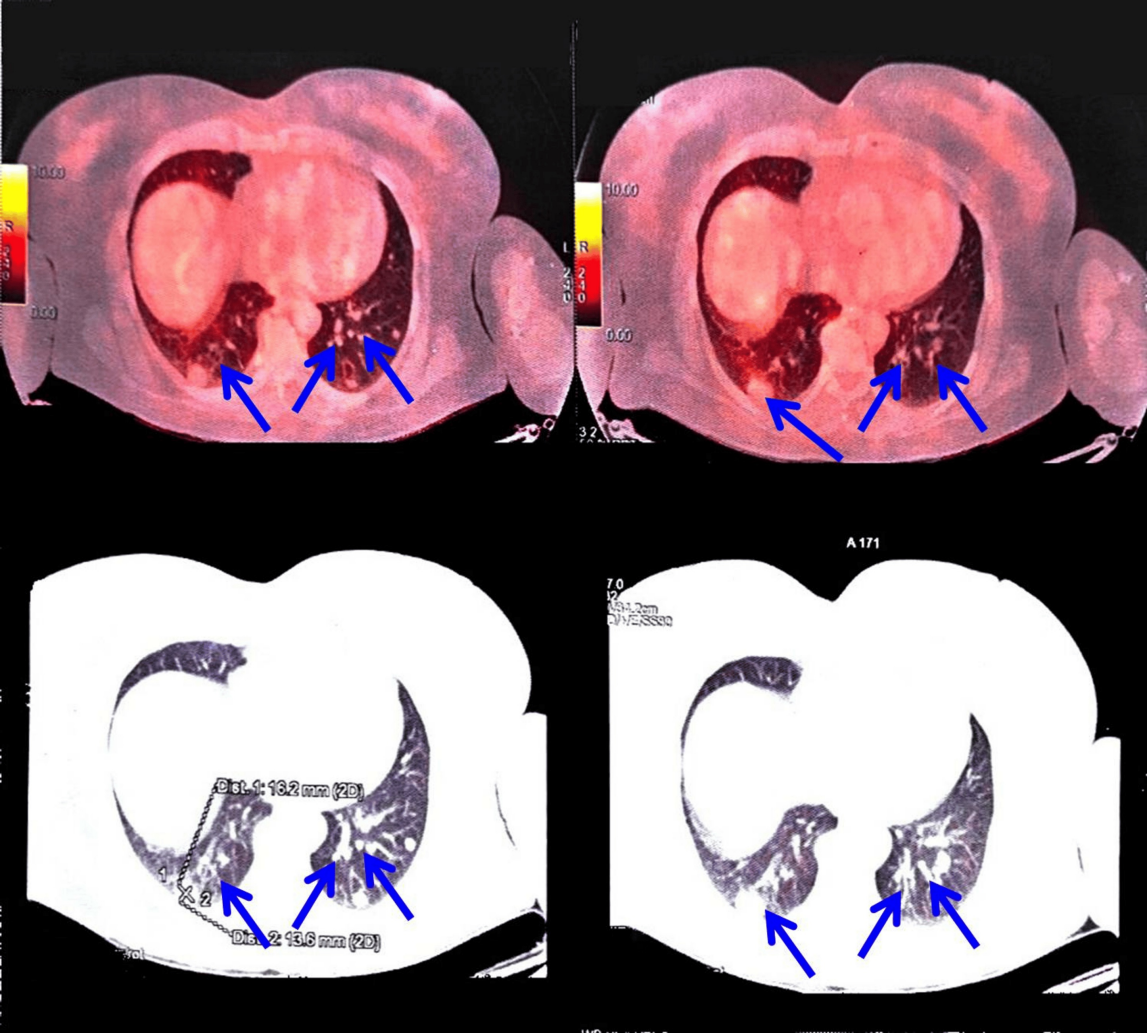
Contrast-enhanced CT chest showing multiple bilateral pleuro-parenchymal nodules Axial contrast-enhanced computed tomography (CT) images of the chest demonstrate multiple bilateral pleuro-parenchymal nodules of varying sizes scattered throughout both lung fields. The largest nodule measures approximately 1.1 cm in diameter. The nodules show well-defined margins without associated consolidation, effusion, or lymphadenopathy, raising clinical suspicion for metastatic or secondary neoplastic involvement during routine follow-up evaluation. The blue arrow indicates the pleuro-parenchymal nodule identified on the contrast-enhanced CT scan.

During her recent follow-up visit, a contrast-enhanced computed tomography (CT) scan of the chest revealed multiple bilateral pleuro-parenchymal nodules of varying dimensions, the largest measuring 1.1 cm in diameter (Figure 1). Given the strong clinical suspicion of metastatic disease, a CT-guided percutaneous core needle biopsy of the largest nodule was performed. Histopathological examination of the biopsy specimen revealed sheets and clusters of tumor cells exhibiting round vesicular nuclei, moderate eosinophilic cytoplasm, and occasional clear-cell changes. A few acinar structures contained eosinophilic secretions. No evidence of increased mitotic activity, necrosis, or hemorrhage was observed, favoring a morphologically benign lesion.

**Figure 2:**
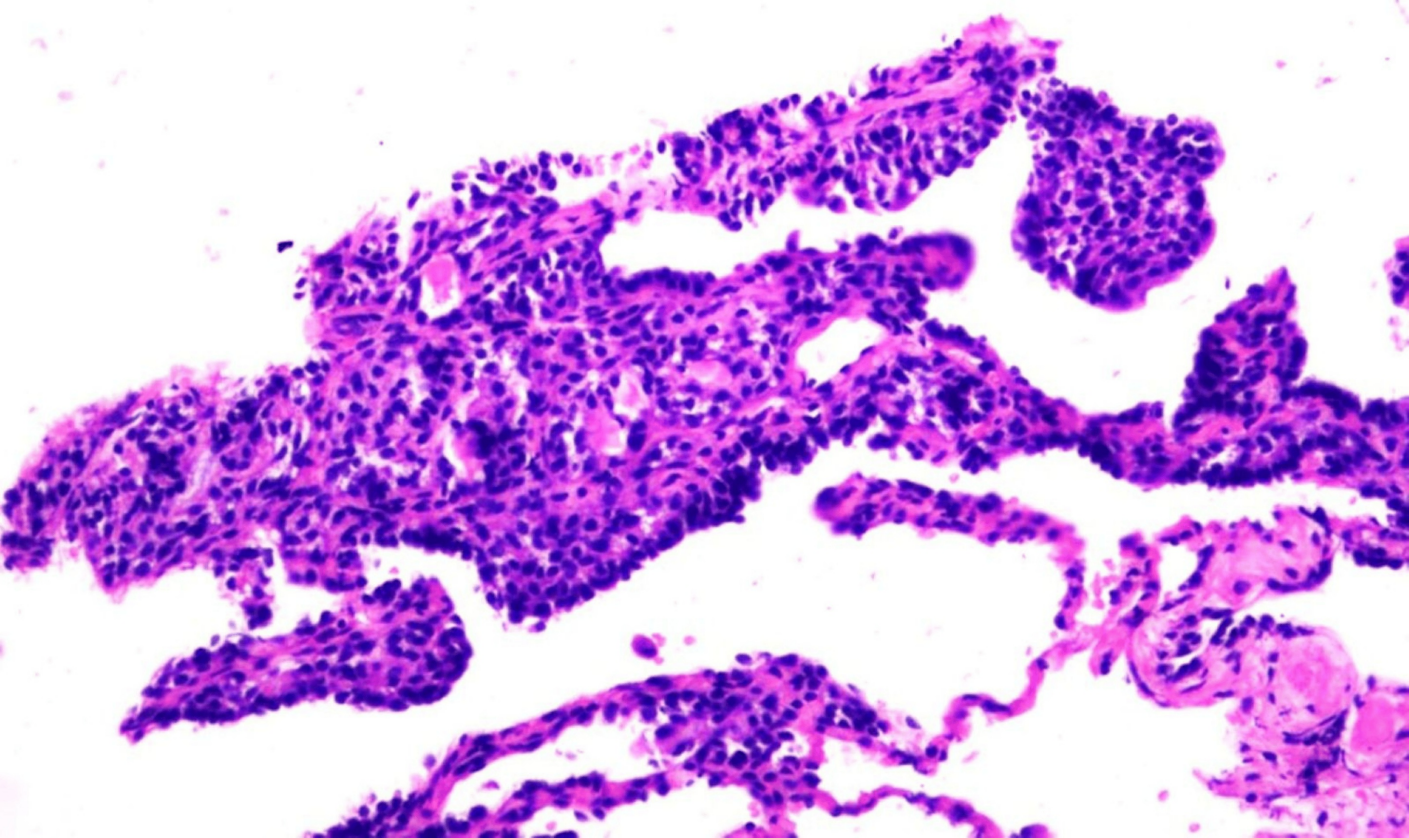
Hematoxylin and eosin (H&E, ×400) H&E, ×400 stained section showing sheets and clusters of tumor cells with round, vesicular nuclei and moderate cytoplasm. Occasional cells exhibit clear cytoplasm, with no evidence of mitosis, necrosis, or hemorrhage, supporting a benign myoepithelial morphology.

**Figure 3:**
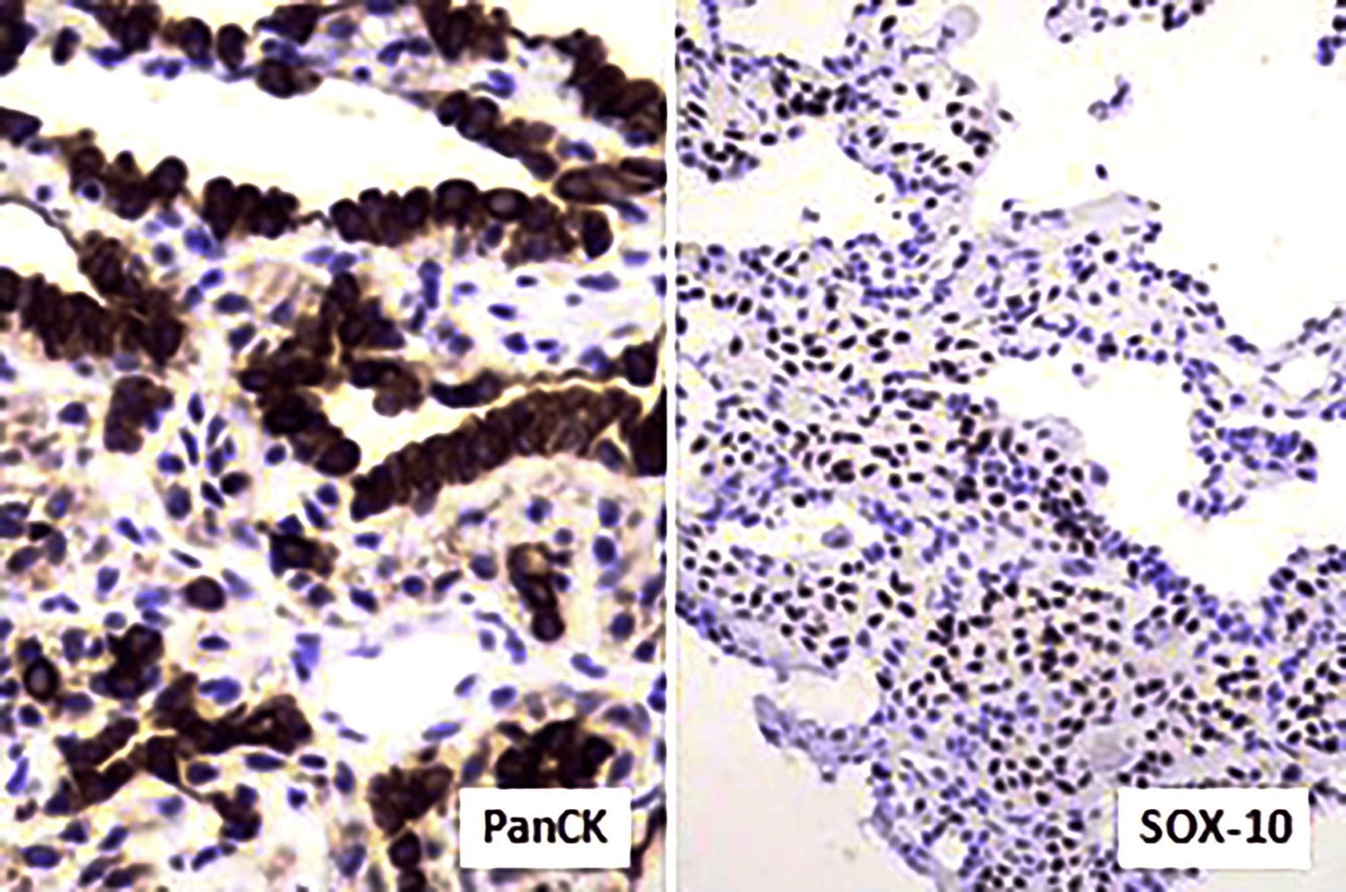
Immunohistochemistry - tumor cells were positive for PanCK and SOX10 Immunohistochemistry demonstrating diffuse cytoplasmic positivity for pancytokeratin (PanCK) and strong nuclear positivity for SRY-box 10 (SOX10) in tumor cells, confirming their epithelial and myoepithelial differentiation. These staining patterns support the diagnosis of benign pulmonary myoepithelioma in correlation with the histomorphological findings.

Immunohistochemical profiling confirmed myoepithelial differentiation, demonstrating diffuse positivity for pancytokeratin (PanCK), smooth muscle actin (SMA), SRY-box 10 (SOX10), p63, and H-Caldesmon. Tumor cells were negative for a comprehensive panel of markers, including S100, thyroid transcription factor-1 (TTF-1), desmin, and melanoma markers, effectively excluding schwannoma, primary pulmonary carcinoma, metastatic sarcoma, and melanoma, among other entities. The Ki-67 proliferation index was <2%, supporting the benign nature of the neoplasm. Based on these histomorphological and immunohistochemical findings, a diagnosis of pulmonary myoepithelioma was established (Figures 2-3). Given the benign behavior of the lesion and the absence of clinical symptoms, no further therapeutic intervention was undertaken, and the patient remains under close clinical and radiological follow-up.

## Discussion

Myoepithelioma of the lung is an exceptionally rare tumor, often detected incidentally on imaging studies performed for unrelated conditions. These tumors typically present as well-circumscribed, slow-growing nodules, and while many remain asymptomatic, some patients may experience cough, dyspnea, or chest discomfort, depending on the tumor size and location. Radiologically, pulmonary myoepitheliomas appear as solitary, well-defined masses, sometimes with calcifications or necrotic areas, and can be mistaken for other benign or malignant lung tumors, such as hamartomas, schwannomas, or primary lung carcinomas [4,5]. Our case featured multiple bilateral pleuro-parenchymal nodules, a presentation highly atypical for a primary benign myoepithelioma and one that initially, and understandably, pointed toward metastatic disease from her prior ACC.

Due to its histopathological and immunohistochemical features, pulmonary myoepithelioma can mimic several other primary and metastatic lung tumors. Pleomorphic adenoma shares histologic similarities with myoepithelioma but contains ductal epithelial components. Immunohistochemically, pleomorphic adenomas express cytokeratins, S-100, and glial fibrillary acidic protein (GFAP), similar to myoepitheliomas, but the presence of glandular differentiation helps distinguish them [6]. Epithelial-myoepithelial carcinoma contains a biphasic pattern of inner ductal epithelial cells and outer myoepithelial cells. Unlike pure myoepithelioma, it demonstrates a dual immunohistochemical profile, with epithelial membrane antigen (EMA)-positive luminal cells and S-100-positive myoepithelial cells [7]. A poorly differentiated lung carcinoma with spindle and pleomorphic cells that may mimic malignant myoepithelioma is sarcomatoid carcinoma, which typically expresses epithelial markers such as cytokeratins and p63, whereas myoepithelioma exhibits myoepithelial differentiation with SMA and S-100 positivity [8]. Schwannoma histologically resembles myoepithelioma due to spindle cell morphology and S-100 positivity. However, schwannomas lack cytokeratin expression, which helps differentiate them from myoepitheliomas [9]. Metastatic myoepithelial tumors from salivary glands or soft tissue may metastasize to the lung, mimicking primary pulmonary myoepithelioma. Clinical history and genetic testing (e.g., Ewing sarcoma breakpoint region 1 (EWSR1) gene rearrangement in some cases) can help distinguish between primary and metastatic disease [10,11]. Due to these overlapping histological and immunohistochemical features, an accurate diagnosis requires a combination of histopathological examination, immunohistochemistry, and clinical correlation.

In the present case, the distinction from a metastasis of the patient’s known ACC was paramount. While both tumors can share myoepithelial features, ACC is characterized by a distinctive cribriform architecture with basophilic mucoid material, which was absent here. Furthermore, the bland cytology, very low Ki-67 index, and pure myoepithelial immunophenotype without a dual cell population are inconsistent with metastatic ACC and definitive for a benign primary pulmonary myoepithelioma [12-14]. In our case, no treatment was administered due to the presence of multiple pleural nodules. The patient remains on regular follow-up and is clinically stable. Prognosis is generally favorable for benign tumors, but malignant variants can exhibit aggressive behavior, including local invasion and distant metastasis. Long-term follow-up is recommended due to the potential for recurrence or malignant transformation [14,15].

Given the scarcity of data on the long-term behavior of multifocal pulmonary myoepithelioma, continued clinical and radiological surveillance is warranted, underscoring the need for further case aggregation to define optimal follow-up protocols.

## Conclusions

This case highlights an exceptionally rare presentation of pulmonary myoepithelioma with bilateral pleural involvement, an unusual presentation that can closely mimic metastatic disease, particularly in patients with a prior history of malignancy. Comprehensive histopathological assessment and targeted immunohistochemistry were pivotal in establishing a definitive benign diagnosis. Recognizing this rare entity is crucial to prevent misdiagnosis, avoid unnecessary interventions, and guide appropriate management. Continued clinical and radiological surveillance remains important due to the limited data or evidence on the long-term outcomes in pulmonary myoepitheliomas.
